# Submesothelial deposition of carbon nanoparticles after toner exposition: Case report

**DOI:** 10.1186/1746-1596-5-77

**Published:** 2010-12-02

**Authors:** Dirk Theegarten, Smail Boukercha, Stathis Philippou, Olaf Anhenn

**Affiliations:** 1 Institute of Pathology and Neuropathology, University Hospital Essen, D-45122 Essen, Germany; 2 Institute of Inorganic Chemistry, University Duisburg-Essen, D-45117 Essen, Germany; 3 Institute of Pathology, Augusta-Hospital, University Duisburg-Essen, D-44791 Bochum, Germany; 4 Department of Pneumology, West German Lung Centre at the University Hospital Essen - University Hospital, University Duisburg-Essen, D-45239 Essen, Germany

## Abstract

Inhalation of carbon nanoparticles (CNP) from toner dust has been shown to have impact on the respiratory health of persons exposed. Office printers are known emitters of CNP. We report about a female open office worker who developed weight loss and diarrhoea. Laparoscopy done for suspected endometriosis surprisingly revealed black spots within the peritoneum. Submesothelial aggregates of CNP with a diameter of 31-67 nm were found by scanning and transmission electron microscopy in these tissue specimens. Colon biopsies showed inflammatory bowel disease with typically signs of Crohn disease, but no dust deposits. Transport of CNP via lymphatic and blood vessels after inhalation in the lungs has to be assumed. In this case respiratory symptoms were not reported, therefore no lung function tests were done. We have shown that workers with toner dust exposure from laser printers can develop submesothelial deposition of CNP in the peritoneum. Impact of toner dust exposure on the respiratory health of office workers, as suspected in other studies, has to be evaluated further.

## Background

Several studies have reported that laser printers are significant sources of ultrafine particles [1-6]. Workers with long time exposure to toner dust showed a significantly higher prevalence of radiographic lung abnormalities in a cross sectional study [7]. Also a significant higher prevalence of temporary coughing and sputum production has been reported [8].

In general nanoparticles (NP) will play a fundamental role in the future and risk assessment seems to be a relevant issue [9]. Office printers were detected to emit carbon nanoparticles (CNP) in a variable extend [5]. Granulomatous pneumonitis and mediastinal lymphadenopathy has been reported in a case of photocopier toner dust exposure [10]. Inhaled ^99m^technetium-labeled CNP can be transported with the human blood circulation and deposited in other organs [11]. We present a female open office worker with toner dust exposure and CNP deposits in the peritoneum.

## Case presentation

A 33-year old female was suffering from intermittent appearing abdominal pain, weight loss and diarrhoea since three months. Biopsies taken during two coloscopies revealed no changes according to the first interpretation at another institution. Therefore her gynecologist suspected endometriosis as a possible cause and admitted her to hospital for laparoscopy. In spite of suspected endometriosis, black spots within the peritoneum were seen and biopsies were taken for histological evaluation. Further history revealed that the patient was working fulltime as an employee in an open-plan office and has been exposed to a laser printer on her personal desk since three years. Up to 70 sheets were printed each working day. Eight laser printers of the same type were installed at other working places in the same office. Respiratory symptoms have not been reported by the patient, therefore lung function tests were not done.

Peritoneal bipsies were fixed in 3.5% buffered formaldehyde and stained conventionally (haematoxylin-eosin, Elastica van Gieson, Prussian blue) for light microscopy (LM). For further analysis of the composition of the black spots formalin fixed paraffin-embedded tissue was cut into slices of 10 μm thickness by a microtome (Microm, Walldorf, Germany), mounted on polyvinylchloride foil and examined by scanning electron microscopy (SEM; ESEM Quanta 400 FEG, FEI, The Netherlands) and energy dispersive X-ray analysis (EDX; EDAX EDS Genesis 4000, Ametek, Germany) as previously described [12]. For analysis of cellular reactions transmission electron microscopy (TEM; Zeiss EM 901A, Oberkochem, Germany) was done using reembedded tissue after adequate processing. Toner material of the office printer was taken for comparison and examined by SEM and EDX.

**Figure 1:**
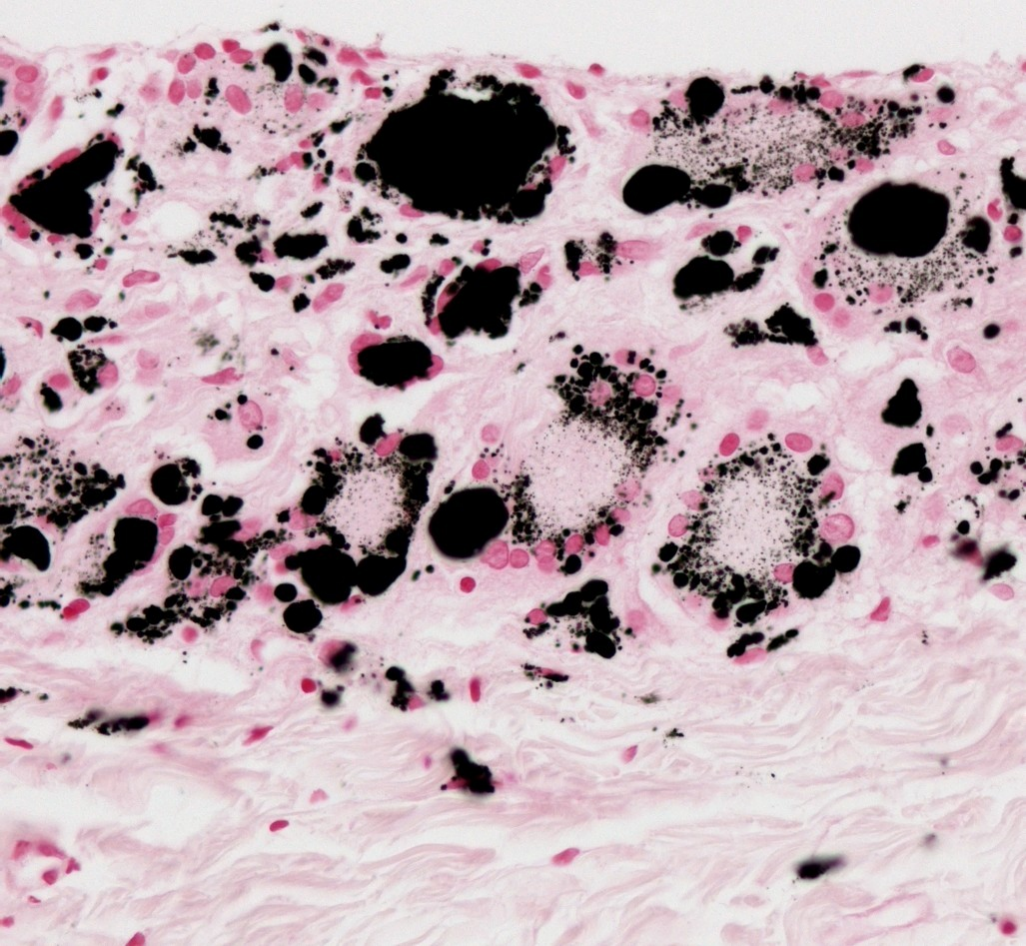
**Light microscopy**. Peritoneal biopsies show black material with a foreign body reaction in submesothelial tissue. Iron positive deposits are not seen (Prussian blue reaction, 400×).

**Figure 2:**
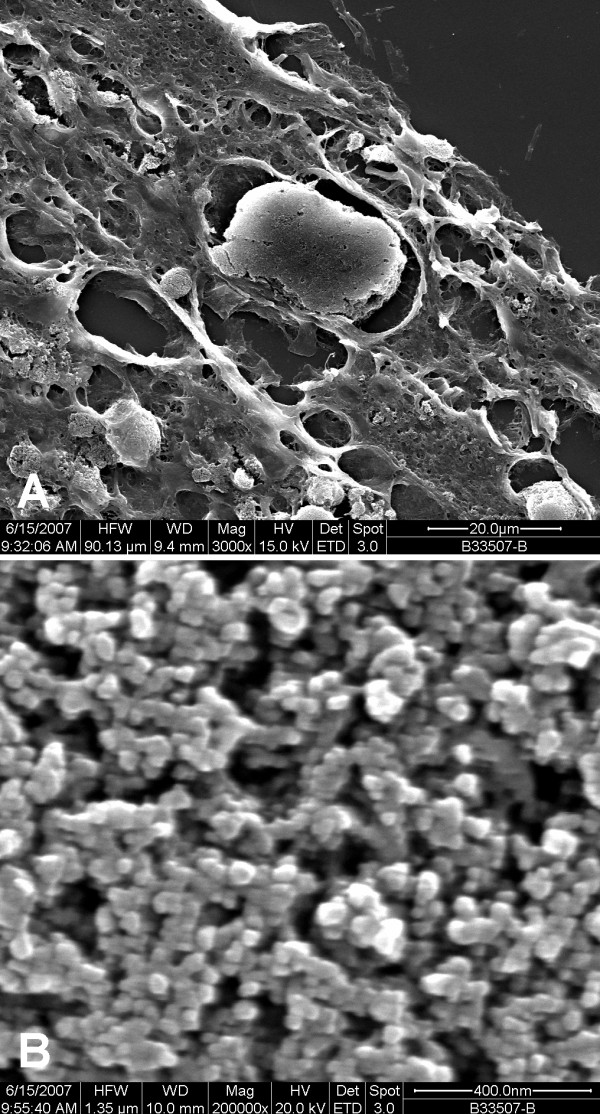
**Scanning electron microscopy**. SEM reveals dense aggregates of foreign material with variable diameters (A, 3,000×), High magnification shows NP with a diameter between 31 and 67 nm (B, 200,000×).

**Figure 3:**
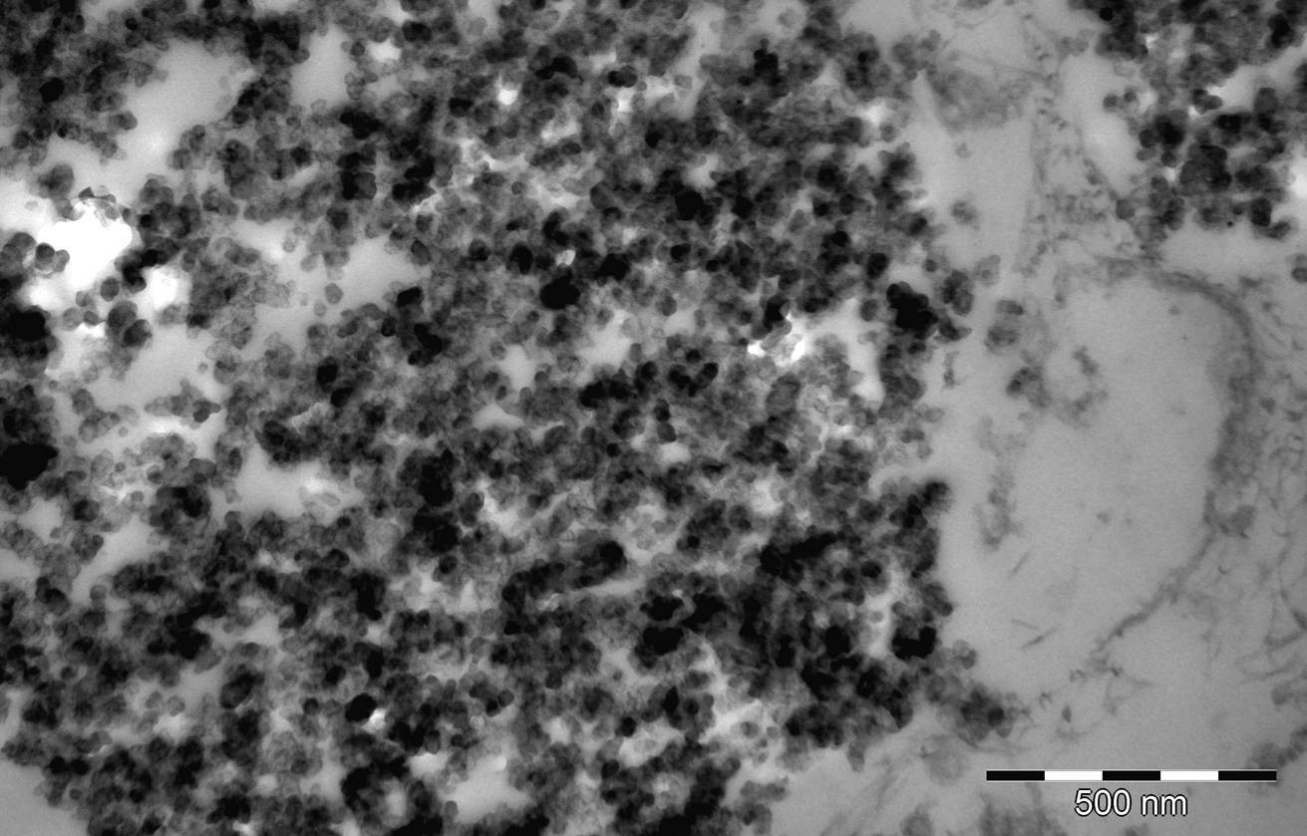
**Transmission electron microscopy**. TEM comfirms NP within phagolysosomes (120,000×).

LM of the peritoneal tissue revealed submesothelial deposits of black material with foreign body reaction (Figure 1). SEM showed submesothelial aggregates of granular material (Figure 2A) consisting of NP with a particle size ranging from 31 to 67 nm (Figure 2B). By EDX no other elements than carbon were found in these aggregates. TEM revealed an inflammatory reaction as in LM. In macrophages phagolysosomes of variable diameters with NP inside were seen (Figure 3). NP showed a similar appearance as in SEM. Toner material was composed of round particles with a diameter of 5-9 μm, with some small elevations on the surface consisting of metal oxides. Because of the lack of endometriosis colon biopsies were reinvestigated by one of the authors. Histological alterations typical for Crohn disease were found and dust deposits were not seen.

## Case discussion

Particle emissions by office printers show differences between printer types, printers of the same type and a significant increase during working times [5]. The average diameter of emitted particles from different printers was found to be in between 40-76 nm and well fitting to our results. The study checked up 62 different printers, but the type used in our case was not included [5]. About a possible respiratory uptake of CNP in human office workers no systematic morphological investigations exist. A study about the respiratory health of workers handling printing toners showed a higher prevalence rate of thoracic radiographic abnormalities and a strong tendency towards a decline of lung function in long time exposed persons [8]. In a case of granulomatous pneumonitis and mediastinal lymphadenopathy with photocopier toner dust exposure containing copper, this metal was detected in the tissue investigated by SEM and EDX [10]. Metal oxides were detectable on the surface of toner particles in our case as well, but deposition in tissue has not been seen. In cases of anthracosilicosis dust deposits in the liver have been reported [13]. This demonstrates that particle transport of inhaled dust via the blood stream with deposition in other organs can be found in humans [11,13]. Ultra fine carbon particles cause a strong down-regulating effect on the cytochrome P450 1B1 protein in monocytes. These data suggest that the induced reduction of gene expression may interfere with the activation and/or detoxification capabilities of inhaled toxic particles. In primary bronchial epithelial cells this effect showed remarkable inter-individual differences, which emphazises the role of polymorphisms [14]. In the case reported here there were no obvious repiratory symptoms. Clinical studies revealed negative effects on respiratory health after toner exposure [7,8,10], therefore further studies concerning morphology, genetics and clinical consequences are needed.

## Conclusion

We have shown that workers with toner dust exposure from laser printers can develop submesothelial deposition of CNP in the peritoneum. Transport of CNP via lymphatic and blood vessels after inhalation in the lungs has to be assumed. Impact of toner dust exposure on the respiratory health of office workers, as suspected in other studies, has to be evaluated further.

## Consent

Written informed consent was obtained from the patient for publication of this case report including images. A copy of the written consent is available for the Editor-in-Chief of this journal.

## List of abbreviations

CNP: carbon nanoparticles; EDX: energy dispersive x-ray analysis; LM: light microscopy; NP: nanoparticles; SEM: scanning electron microscopy; TEM: transmission electron microscopy

## Competing interests

The authors declare that they have no competing interests.

## Authors' contributions

DT did LM of the peritoneum (second opinion) and TEM, supervised SEM and EDX and wrote the manuscript. SB performed SEM and EDX, SP has done LM of the peritoneum and colon and initiated further investigations. OA discussed the clinical background and revised the manuscript. All authors read and approved the final version.
